# Health Seeking Behavior among Rural Left-Behind Children: Evidence from Shaanxi and Gansu Provinces in China

**DOI:** 10.3390/ijerph15050883

**Published:** 2018-04-28

**Authors:** Hongyu Guan, Huan Wang, Juerong Huang, Kang Du, Jin Zhao, Matthew Boswell, Yaojiang Shi, Mony Iyer, Scott Rozelle

**Affiliations:** 1 Center for Experimental Economics in Education, Shaanxi Normal University, Xi’an, 710127, China; guanhongyu2016@163.com (H.G.); huangjuerongceee@163.com (J.H.); dukangceee@163.com (K.D.); zhaojinceee@163.com (J.Z.); shiyaojiang7@gmail.com (Y.S.); rozelle@stanford.edu (S.R.); 2 Freeman Spogli Institute for International Studies, Stanford University, Stanford, CA 94305, USA; kefka@stanford.edu; 3 Onesight Foundation, 4000 Luxottica Pl, Mason, OH 45040, USA; viyer@onesight.org

**Keywords:** randomized controlled trial, rural China, left-behind children, healthcare

## Abstract

More than 60 million children in rural China are “left-behind”—both parents live and work far from their rural homes and leave their children behind. This paper explores differences in how left-behind and non-left-behind children seek health remediation in China’s vast but understudied rural areas. This study examines this question in the context of a program to provide vision health care to myopic rural students. The data come from a randomized controlled trial of 13,100 students in Gansu and Shaanxi provinces in China. The results show that without a subsidy, uptake of health care services is low, even if individuals are provided with evidence of a potential problem (an eyeglasses prescription). Uptake rises two to three times when this information is paired with a subsidy voucher redeemable for a free pair of prescription eyeglasses. In fact, left-behind children who receive an eyeglasses voucher are not only more likely to redeem it, but also more likely to use the eyeglasses both in the short term and long term. In other words, in terms of uptake of care and compliance with treatment, the voucher program benefitted left-behind students more than non-left-behind students. The results provide a scientific understanding of differential impacts for guiding effective implementation of health policy to all groups in need in developing countries.

## 1. Introduction

More than 60 million children in rural China are “left-behind”—both parents live and work far from their rural homes and leave their children behind [1]. The left-behind phenomenon is driven in large part by China’s rapid development and urbanization, and in particular by the migration of large numbers of rural residents from their rural homes to urban areas in search of better job opportunities [2]. It is common for migrant parents to leave their children behind with a caregiver—typically the paternal grandparents—in their home communities [1,3,4]. This has created a new, large, and potentially vulnerable subpopulation of left-behind children in rural areas.

There is great concern about whether left-behind status disproportionately hurts children’s health and well-being. Previous research suggests that health outcomes are worse for left-behind children than for children whose parents are at home [5,6,7,8,9]. Additionally, left-behind children may be at greater risk of depression, anxiety, and loneliness as a result of separation from their parents [5,8,10,11,12,13,14]. Compared with children living with both parents, left-behind children are also reported to have poorer physical development (e.g., higher rates of stunting and wasting), lower nutrition and higher rates of anemia [6,7].

However, the reasons why left-behind children may have poorer health than their non-left-behind peers is not well studied. One possible mechanism for lower health outcomes is that elderly surrogate caregivers may not be as proactive as parents in seeking health remediation for children. This may be especially true when considering health conditions that are not acute or have few obvious symptoms, but for which treatment can yield important gains in wellbeing and/or productivity [3,15]. For such conditions, preemption on the part of caregivers may factor highly in uptake of remediation.

Little evidence exists to substantiate the difference in healthcare-seeking behaviors between left-behind children and children whose parents are at home (hereafter, left-behind and non-left-behind families). Particularly, little is known about how left-behind families respond to the healthcare needs of children in comparison to caregivers of non-left-behind children. Some research suggests that grandparents may be less likely to help children in need because they grew up decades earlier and may be less inclined to understand health risks and resources [16]. Systematic differences in health seeking behavior between working-age and elderly caregivers also may occur on account of variations in the availability of time, liquidity constraints, access to and availability of information, or frequency of visits to areas where healthcare services are present [17,18,19,20,21]. In rural China, distances to the nearest county seat, which is often where reliable service is based, can involve hours of travel, potentially affecting the decision to seek care and the actual uptake of care for older caregivers [22,23]. To the extent that families of left-behind children behave differently in seeking healthcare remediation, targeted policies may be needed to reach this important subgroup.

A series of studies suggest that approximately 10 to 15% of school-aged children in the developing world have common vision problems [24,25,26,27]. In most cases children’s vision problems can be easily corrected by timely and proper fitting of quality eyeglasses [28]. Unfortunately, studies in a variety of developing countries document that 35 to 85% of individuals with refractive errors do not have eyeglasses [29,30,31].

In rural areas of China the prevalence of vision problems among children is among the highest in the world [27,32,33]. One recent study in the same area as our study shows that about 25% of students in grades 4 and 5 have myopia [31]. However, recent investigations in rural China demonstrate that fewer than one third of children needing glasses actually have glasses and even fewer wear them [34]. According to Yi et al. [31], more than 85% of children in rural China with myopia do not wear glasses.

For myopic children, wearing eyeglasses has substantial benefits. Beyond the improvement in quality of life due to improved vision, providing children with glasses has been shown to significantly improve academic performance [33,35]. The effect of glasses on the schooling outcomes of students is estimated to be at least as large as that of well-known education interventions deemed highly successful [36].

Several factors contribute to the high rates of uncorrected vision problems found in these studies. Research suggests that the lack of awareness about the importance of wearing glasses and misinformation contribute to low usage rates in China [34]. For example, there are commonly held (but mistaken) views in many countries, including China, that wearing eyeglasses will harm one’s vision [31,34].

Due to such misinformation and limited access to screenings and exams, eyeglasses wear remains limited in China [37]. Specifically, rural students and their families may not know how to go about getting their first pair of glasses. As a consequence, it is possible that a well-run government program that subsidized vision care services for youth might be needed.

The overall objective of this study is to understand variations in how left-behind and non-left-behind families seek health remediation.

To meet this objective, the study examines this question in the context of an in-the-field randomized controlled experiment seeking to determine the effect of a program to provide subsidized eyeglasses to myopic children. The main results of the study are reported in Ma, et al. 2014, and the Ma et al. paper examine program impacts on eyeglasses uptake and usage, as well as schooling outcomes for children [33,38]. The intervention which subsidizes vision care is a useful setting to study differential health seeking behaviors between left-behind and non-left-behind families because preemptive behavior on the part of caregivers, rather than a response to obvious symptoms in the child, may be a primary driver of timely remediation.

In carrying out a sub-group analysis of differential responses between left-behind children and non-left behind children, the current study focuses on three primary factors. First, the analysis determines to what extent families, irrespective of left-behind status, seek vision care when they are made aware that their child may suffer from a vision problem. Second, the paper explores whether a voucher subsidy designed to boost uptake of care differentially affects left-behind and non-left-behind family subgroups. In approaching this second factor, the analysis compares both the uptake of care and the usage of eyeglasses over the short and longer term. Finally, the study analyzes how distance to the care provider (a proxy for the cost of seeking health care) may affect uptake of vision care service across left-behind and non-left-behind subgroups, both with and without a voucher subsidy. In these ways, the study aims to understand how health interventions can mediate differences in health seeking behavior as they relate to left-behind and non-left-behind families.

The remainder of the paper is organized as follows: Section 2 describes the experiment and data collection. Section 3 presents the results. Section 4 and Section 5 discuss the policy implications of the experimental results and concludes.

## 2. Materials and Methods

### 2.1. Study Area and Sampling Technique

The experiment from which the data in this paper are taken took place in two adjoining provinces of western China: Shaanxi and Gansu. These two provinces have a total population of 65 million, and can reasonably represent poor rural areas in northwest China. In 2012, Shaanxi’s gross domestic product per capita of USD 6108 was ranked 14th among China’s 31 provincial administrative regions, and was similar to that for the country as a whole (USD 6091) in the same year. Gansu’s gross domestic product per capita, USD 3100, makes Gansu the second-poorest province in the country according to China National Statistics Yearbook in 2012.

In each of the provinces, one prefecture (each containing a group of seven to ten counties) was included in the study. The prefectures are fairly representative of the provinces. The prefecture in Gansu has a population of 3.3 million making up 10% of the provincial population. The GDP per capita of USD 2680 is very close to the provincial level. The prefecture in Shaanxi has a population of 3.4 million, about 10% of the provincial population. While GDP per capita is USD 13,100, higher than the provincial average, in no small part this is due to mining and other extractive industries; income per capita is close to the provincial average.

To choose the sample, we obtained a list of all rural primary schools in each prefecture. To minimize the possibility of inter-school contamination, we first randomly selected 167 townships and then randomly selected one school per township for inclusion in the experiment. Within schools, our data collection efforts (discussed below) focused on grade 4 and grade 5 students. From each grade, one class was randomly selected and surveys and visual acuity examinations. More than 95 percent of poor vision is due to myopia. For simplicity, we will use myopia to refer to vision problems more generally.

### 2.2. Data Collection

#### 2.2.1. Baseline Survey

A baseline survey was conducted in September 2012. The baseline survey collected detailed information on schools, students and households. The school survey collected information on school infrastructure and characteristics (including distance to county seat). A student survey was given to all students in selected grade 4 and grade 5 classes. The student survey collected information on basic background characteristics, including age, gender, whether boarding at school, whether they owned eyeglasses before, and their knowledge of vision health. Students were also asked about whether their parents worked away from home for more than six months per year. Students indicating this to be the case for both parents were defined as left-behind children for the purpose of our analysis. Students that reported one or both parents at home for more than 6 months a year were considered non-left-behind children. Household surveys were also given to all students, which they took home and filled out with their caregivers. The head teacher of each classroom collected the completed household forms and forwarded them to the survey team. The household survey collected information on households that children would likely have difficulty answering (e.g., parents’ education levels and the value of family assets).

#### 2.2.2. Vision Examination

At the same time as the school survey, a two-step vision examination was administered to all students in the randomly selected classes in all sample schools. First, a team of two trained staff members administered visual acuity screenings using Early Treatment Diabetic Retinopathy Study (ETDRS) eye charts, which are accepted as the worldwide standard for accurate visual acuity measurement [39]. Students who failed the visual acuity screening test (cutoff was defined by visual acuity of either eye less than or equal to 0.5, or 20/40) were enrolled in a second vision test that was carried out at each school one or two days after the first test. 

The second vision test was conducted by a team of one optometrist, one nurse and one staff assistant and involved cycloplegic automated refraction with subjective refinement to determine prescriptions for students needing glasses. These are standard procedures when conducting a vision exam for children to prescribe eyeglasses. Cycloplegia refers to the use of eye drops to briefly paralyze the muscles in the eye that are used to achieve focus. The procedure is commonly used during vision exams for children to prevent them from reflexively focusing their eyes and rendering the exam inaccurate. It was after the exam that prescriptions and (in the case of the Voucher group) vouchers and instructions were given to the students to take home to their families.

In order to calculate and compare different visual acuity levels we require a linear scale with constant increments [40,41]. In the field of ophthalmology/optometry LogMAR is one of the most commonly used continuous scales. This scale uses the logarithm transformation: e.g., LogMAR = log10 (MAR). In this definition, the variable, MAR, is short for Minimum Angle of Resolution, which is defined as the inverse of visual acuity, e.g., MAR = 1/VA. LogMAR offers a relatively intuitive interpretation of visual acuity measurement. It has a constant increment of 0.1 across its scale; each increment indicates approximately one line of visual acuity loss in the ETDRS chart. The higher the LogMAR value, the worse one's vision is. 

#### 2.2.3. Eyeglasses Uptake and Usage

Our analysis focuses on two key variables: eyeglasses uptake rate and usage rate. A short-term follow up survey was done in early November 2012 (one month after vouchers were distributed). A long-term follow up survey was conducted in May 2013 (seven months after vouchers were distributed).

Uptake in our study is defined by ownership. Specifically, we define uptake as a binary variable taking the value of one if a student owns a pair of eyeglasses at baseline or acquired one during the program (regardless of the source). Students that had been diagnosed with myopia in baseline vision examination were given a short survey that included questions about whether they owned eyeglasses and how they acquired them. If a student indicated he or she did have glasses but was not wearing them we confirmed by asking to see them. If the eyeglasses were at home, we followed up with phone calls to the caregivers.

Usage is a binary variable measured by students’ survey responses of whether students wear eyeglasses regularly when they study and outside of class. To reduce reporting bias, a team of two enumerators made unannounced visits to each of the 167 schools in advance of the long term follow up survey. During the unannounced visits, enumerators were given a list of the students diagnosed with myopia in the baseline and record individual-level information on their usage of glasses. We double-checked student responses with our unannounced visits data. The significantly correlation suggesting the student responses data are reliable.

### 2.3. Experimental Design

Following the baseline survey and vision tests (described above), schools were randomly assigned to one of two groups: Voucher or Prescription. Details of the nature of the intervention in each group are described below. To improve power, we stratified the randomization by county and by the number of children in the school found to need eyeglasses. In total, this yielded 45 strata. Our analysis takes this randomization procedure into account [42]. The trial was approved by Stanford University (No. ISRCTN03252665, registration site: http:// isrctn.org).

The two groups were designed as follows:

Voucher Group: In the Voucher group, each student diagnosed with myopia was given a voucher as well as a letter to their parents informing them of their child’s prescription. Details on the diagnosis procedures are provided in the Data Collection subsection below. The vouchers were non-transferable. Information identifying the student, including each student’s name, school, county, and student’s prescription was printed on each voucher and students were required to present their identification in person to redeem the voucher. This voucher was redeemable for one pair of free glasses at an optical store that was in the county seat. Program eyeglasses were pre-stocked in the retail store of one previously-chosen optometrist per county. The distance between each student’s school and the county seat varied substantially within our sample, ranging from 1 km to 105 km with a mean distance of 33 km. While the eyeglasses were free, the cost of the trip in terms of time and any cost associated with transport were born by family of the student.

**Figure 1:**
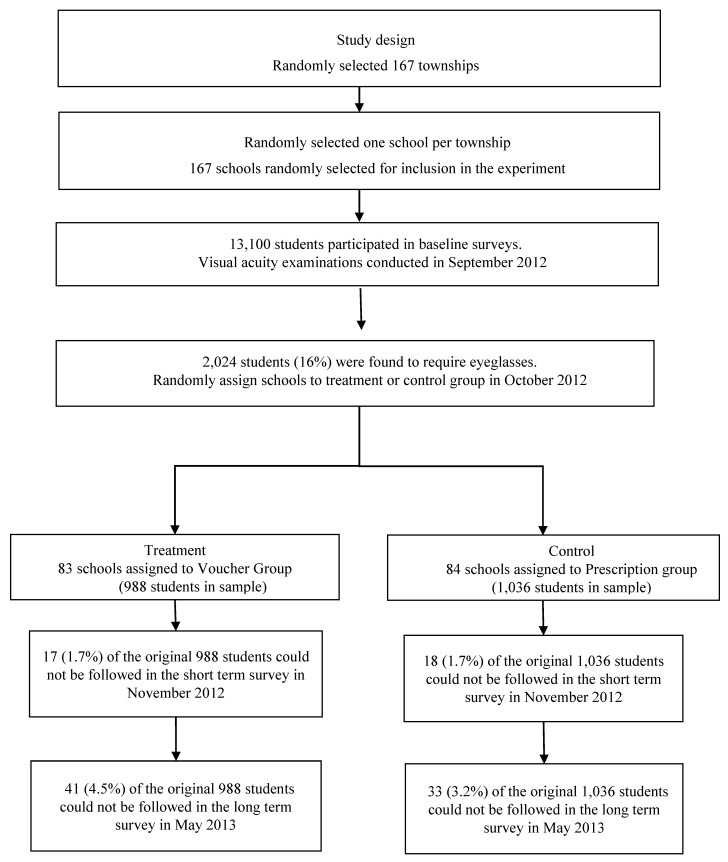
Schematic diagram describing the sample for the study

Prescription Group: Myopic students in the Prescription group were given a letter for their parents informing them of their child’s myopia status and prescription. No other action was taken. If they opted to purchases glasses, they would also have to travel to the county seat. Unlike the families in the Voucher group, the families in the Prescription group would have to select an optical shop and purchase the eyeglasses. Figure 1 shows the research design of this study.

### 2.4. Tests for Balance and Attrition Bias

Of the 13,100 students in 167 sample schools who were given vision examinations at baseline, 2024 (16%) were found to require eyeglasses. Only these students are included in the analytical sample. There were 988 students in 83 sample schools that were randomly assigned to the Voucher group and 1036 students in 84 sample schools that were randomly assigned to the Prescription group.

Table 1 shows the balance check of baseline characteristics of the students in our sample across experimental groups. The first column shows the mean and standard deviation in the Prescription group. Column 2 shows the mean and standard deviation in the Voucher group. We then tested the difference between students in the Prescription and Voucher groups adjusting for clustering at the school level. The results in column 3 suggest a high level of overall balance across the two experimental groups. There is no significant different between the two groups in student age, gender, boarding status, grade, ownership of eyeglasses, belief that eyeglasses will harm vision, visual acuity, parental education, family member eyeglasses ownership, family assets, distance from school to county seat, and parental migration status. 

Irrespective of left-behind status, only 14% of students who needed glasses had them at the time of the baseline (row 5). Fifty percent of students in the sample lived with both parents at home (row 13). About 12% of students had parents that both migrated elsewhere for work and were consider left-behind children (row 14). The other 38 percent of the children in our sample lived with one parent, either their father or mother.

Attrition at the short- and long-term visits was limited (Figure 1). Only 35 (1.7%) out of 2024 students could not be followed up with in the short term and 74 (3.6%) could not be followed up with in the long term. Due to attrition, the sample of students for whom we have a measure of eyeglasses uptake and usage in the short term and long term is smaller than the full sample of students at the baseline. We therefore tested whether the estimates of the impacts of providing voucher on eyeglasses uptake and usage are subject to attrition bias. To do so, we first constructed indicators for attrition at the short term or long term (1 = attrition). We then regressed different baseline covariates on a treatment indicator, the attrition indicator (one for the short term and long term, respectively), and the interaction between the two. We also adjusted for clustering at the school level. The results are shown in Table 2. Overall, we found that there were no statistically significant differences in the attrition patterns between treatment groups and control groups on a variety of baseline covariates as of the short term and long term. There is only one exception. The short-term survey results showed that compared with attritors in the Prescription group, attritors in the Voucher group were less likely to believe that eyeglasses can harm vision (Table 2, panel A, row 3, column 6). This difference is significant at the 5% level.

### 2.5. Statistical Approach

Unadjusted and adjusted ordinary least squares (OLS) regression analysis are used to estimate how eyeglasses uptake and usage changed for children in the Voucher group relative to children in the Prescription group. We estimate parameters in the following models for both the short and long term. The basic specification of the unadjusted model is as follows:

(1) $$y_{\mathrm{ijt}}=\alpha +\beta_{V}{\mathrm{Voucher}}_{j}+\varepsilon_{\mathrm{ijt}},$$

where $y_{\mathrm{ijt}}$ is a binary indicator for the eyeglass uptake or eyeglasses usage of student i in school j in wave t (short-term or long-term). ${\;\mathrm{Voucher}}_{j}$ is a dummy variable indicating schools in the Voucher group, taking on a value of 1 if the school that the student attended was assigned to Voucher group and 0 if the school that the student attended was in the Prescription group. $\varepsilon_{\mathrm{ijt}}$ is a random error term.

To improve efficiency of the estimated coefficient of interest, we also adjusted for additional covariates (X_ij_). We call Equation (2) below our adjusted model:

(2) $$y_{\mathrm{ijt}}=\alpha +\beta_{V}{\mathrm{Voucher}}_{j}+{Χ}_{\mathrm{ij}}+\varepsilon_{\mathrm{ijt}},$$

The additional X_ij_ represents a vector of student, family, and school characteristics. These characteristics including student’s age in years, whether student is male, whether the student is boarding at school, whether student is in 4th grade, whether student owns eyeglasses at baseline, whether student thinks that eyeglasses will harm vision, severity of myopia measured by the LogMAR. The family characteristics include dummy variables for whether parents have high school or above education, whether a family member wears eyeglasses, household asset index calculated using a list of 13 items and weighting by the first principal component. Finally, X_ij_ also includes the distance from the school to the county seat, and whether the student is left-behind child. 

To analyze the paper’s main question of interest—whether the Voucher intervention affected left-behind children differently, we estimate parameters in the following heterogeneous effects model:

(3) $$y_{\mathrm{ijt}}=\alpha +\beta_{V}{\mathrm{Voucher}}_{j}+\beta_{L}{\mathrm{Leftbehind}}_{i}+\beta_{\mathrm{VL}}{\mathrm{Voucher}}_{j}\times {\mathrm{Leftbehind}}_{i}+{Χ}_{\mathrm{ij}}+\varepsilon_{\mathrm{ijt}},$$

where ${\mathrm{Leftbehind}}_{i}$ is a dummy variable indicating that a student is a left-behind child. The coefficient $\beta_{V}$ compares eyeglasses uptake or usage in the Voucher group to that in the Prescription group; $\beta_{L}$ captures the effect of being a left-behind child on eyeglasses uptake or usage. The coefficients on the interaction terms $\beta_{\mathrm{VL}}$ give the additional effect (positive or negative) of the voucher on eyeglasses uptake or usage for the left-behind children relative to the voucher effect for the non-left-behind children.

In all regression models, we adjust standard errors for clustering at the school level using the cluster-corrected Huber-White estimator. To understand the relationship between distance and the utilization of healthcare, a nonparametric Locally Weighted Scatterplot Smoothing (Lowess) plot is used to the illustrate the eyeglasses uptake rates and the distance from school to county seat (where students redeem voucher for eyeglasses). One advantage of using the Lowess approach is that it can provide a quick summary of the relationship between two variables [43,44].

## 3. Results

This section reports the four main findings of the analysis. First part reports the levels of eyeglasses usage at the time of baseline (before any interventions occurred) among both left-behind and non-left-behind students. Second part estimates the average impact of providing a subsidy Voucher on student eyeglasses uptake and usage. Third part looks at the heterogeneous effects of the voucher on left-behind children versus their non-left-behind peers. Last part reports the extent to which distance to the county seat (where service is available) affects eyeglasses uptake in both the Voucher and Prescription groups.

### 3.1. Eyeglasses Usage at the Time of Baseline among Left-Behind and Non-Left-Behind Students with Poor Vision

Table 3 shows differences in left-behind children and non-left-behind children with poor vision at the time of baseline. Across most of the control variables the two types of students are the same. Exceptions include a slightly higher level (6.9%age points) of education among the mothers of left-behind children (Table 3, column 3, row 9, significant at the 1% level) and a higher level of household assets among non-left-behind children (column 3, row 11, significant at the 1% level). Uptake and usage of eyeglasses at the time of baseline is the same between the two subgroups and quite low, about 13% (column 3, row 5). Nearly 40% of students believed that wearing eyeglasses would negatively affect one’s vision (row 6). Generally, the parental education level was low: 15% of students’ fathers and less than 10% of the mothers had attended high school (row 9).

### 3.2. Average Impacts of Providing Voucher on Student Eyeglasses Uptake and Usage

The results for average impact on eyeglasses uptake and usage (irrespective of left-behind status) are shown in Table 4. Columns 1 to 4 show estimates for eyeglasses uptake, columns 5 to 8 show the results for eyeglasses usage. Odd-numbered columns show the results from unadjusted model (Equation (1)) and even-numbered columns show the results from adjusted model (Equation (2)). The estimates from both unadjusted model and adjusted model in are included in Table 4 for short term (one month after vouchers were distributed) and long term (seven months after vouchers were distributed).

The results show that the eyeglasses uptake rate in the Voucher group is significantly higher than in the Prescription group in both short term and long term. In the unadjusted model, at the time of the short-term check, the average eyeglasses uptake rate among children that received only a prescription was about 25% (Table 4, column 1, last row). The uptake rate among children in the Voucher group was 85%, more than three times higher than the Prescription group. At the time of the long term, the average eyeglasses uptake rate among Prescription group was about 43% (column 3, last row), compared to 87% uptake rate among Voucher group. The adjusted model results, which control for student characteristics, and family characteristics, suggest a similar story: Voucher treatment yields positive impacts over both the short and long term. Specifically, our adjusted results show that the eyeglasses uptake rate in the Voucher group was 61 percentage points higher at the time of short term survey (row 1, column 2) and 45 percentage points higher at long term survey (column 4) than the Prescription group. These results are all significant at the 1% level.

Moreover, the results reveal that the eyeglasses usage in Voucher group is significantly higher in both short term and long term. The unadjusted model results show that in the short term, the percentage of students who use eyeglasses was 65% in the Voucher group—more than three times higher than the 21 percent usage in the Prescription group (Table 4, column 5, last row). The long-term results show that the percentage of students who use eyeglasses was 35% in the Prescription group (column 7, last row), compared with 59 percent in the Voucher group. In the adjusted model, our results also show that the Voucher increased eyeglasses usage by 45 percentage points in the short term (column 6, row 1) and 26 percentage points in the long term (column 8, row 1). These results are also significant at the 1% level.

### 3.3. Heterogeneous Effects on Left-Behind Children

This section examines the main question of interest in the paper. The results indicate that providing the subsidy Voucher on average has a consistent positive impact on eyeglasses uptake and usage in both short term and long term, but there is substantial differential impact between left-behind and non-left-behind children.

As shown in Table 5, in the Prescription group uptake and usage rates among left-behind children were much lower than among their non-left-behind peers. Specifically, the results show that in the short term, the left-behind children were 8 percentage points less likely to purchase eyeglasses than non-left-behind children (Table 5, columns 1 and 2, row 2). This is significant at the 10% level. In the long term, the left-behind children were also 10 percentage points less likely to purchase eyeglasses (columns 3 and 4, row 2). This is significant at the 5% level. There also was no significant difference in eyeglasses usage between left-behind children and non-left-behind children in the short term (columns 5 and 6, row 2). However, in the long term, the left-behind children were 14 percentage points less likely to use the eyeglasses than their peers (columns 7 and 8, row 2). These results are significant at the 5% level.

Although left-behind students in the Prescription exhibit lower rates of both uptake and usage when compared to their non-left-behind peers, in the voucher group the trend was almost reversed. The results show that in the Voucher group, the eyeglasses uptake rates among left-behind children were higher than among their non-left-behind peers in both the short term and long term. Specifically, the coefficients for the interaction term between Voucher group and whether the student is a left-behind child are all positive and statistically significant (Table 5, columns 1 through 4, row 3). The eyeglasses uptake rate among left-behind children in the intervention group was additional 8.4 to 6.8 percentage points higher by short term survey (significant at the 1 percent level—columns 1 and 2, row 3). More importantly, the effect was sustained over time. At the time of the long-term survey, the eyeglasses uptake rate among the left-behind children in the Voucher group continued to be additional 13.1 to 11.8 percentage points higher than the non-left-behind children (significant at the 1% level—columns 3 and 4, row 3).

In terms of eyeglasses usage, heterogeneous effect results show that the left-behind children in the Voucher intervention group had a higher (but insignificant) usage rate in the short term, and also had a higher (and significant) usage rate in the long term (Table 5, row 3, columns 5 to 8). The eyeglasses usage rates among left-behind children in the Voucher group increased by an additional 7.6 and 7.2 percentage points by our short-term survey, however this is statistically insignificant (row 3, columns 5 and 6). By the long term, the eyeglasses usage among the left-behind children in the Voucher intervention group was 14.6 and 13.7 percentage points higher than the non-left-behind children (significant at the 1% level—row 3, columns 7 and 8).

Differential impacts on usage across left-behind and non-left-behind families are also instructive. Signals highlighting the importance of the subsidized health service may be affecting elderly caregivers more than working age parents at home. If elderly caregivers respond to a possible signal by increasing their perceived utility of treating poor vision for their dependents, they may supervise glasses wear with more rigor than parents whose value of the treatment could be crowded out by other concerns.

### 3.4. The Relationship between Distance and the Utilization among Rural Families

The hypothesis that time is less valuable among elderly caregivers is borne out by an analysis of distance from service providers and uptake of care. In the discussion above we find that the Voucher intervention not only had a significant average impact on eyeglasses uptake and usage, but also had an additional impact on the left-behind children. In this section examines how “distance from the county seat” (or the cost of using the Voucher) affects the impact across the different sub-groups. To do so, an exploratory analysis is conducted to compare the demand curves of the left-behind children and with non-left-behind children.

**Figure 2:**
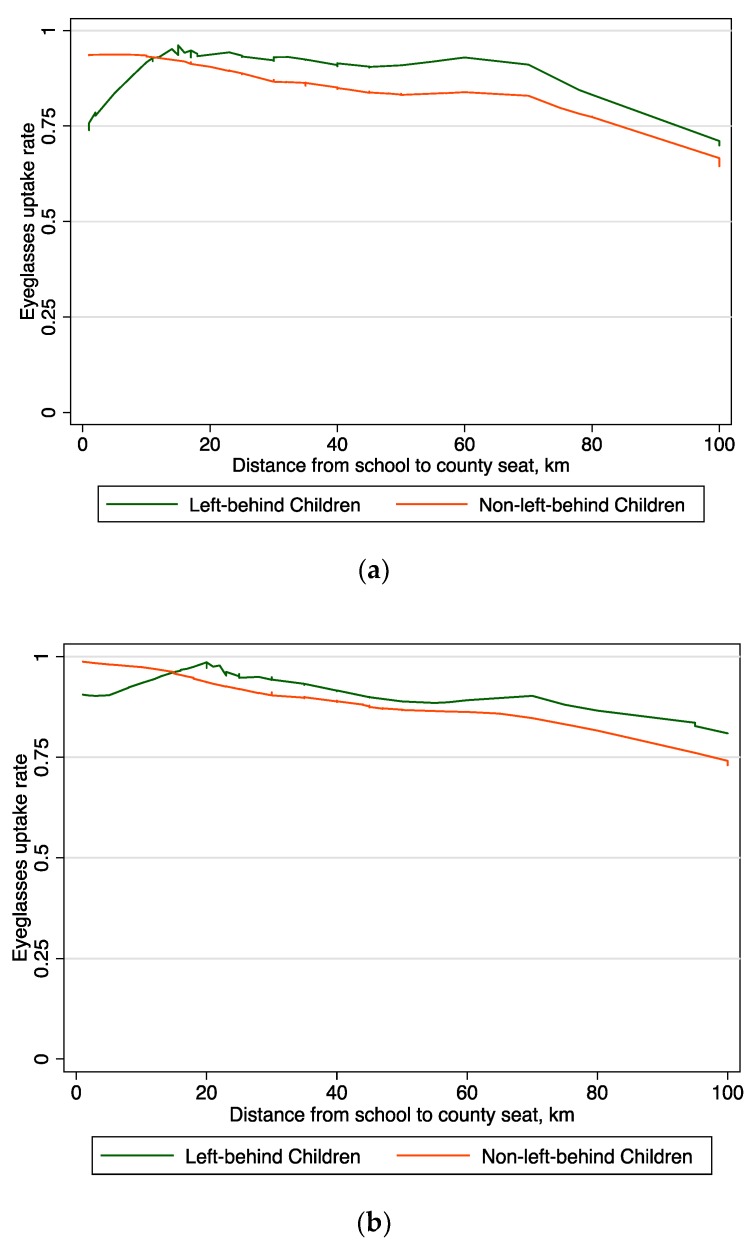
Lowess Plot of eyeglasses uptake rates and distance to county seat among voucher group. (**a**) One month following voucher distribution; (**b**) seven months following voucher distribution.

The Lowess plots among the Voucher group show that the left-behind children’s eyeglasses uptake rate is higher than the non-left-behind children, meaning the left-behind children were more likely to redeem their vouchers than the non-left-behind children in both short term and long term (Figure 2a,b). Although the plots demonstrate a quick summary of the generally negative relationship between eyeglasses uptake rates and distance to the county seat, as the distance move from left to right across the graph, the eyeglasses uptake rate for the left-behind children falls more gradually than that of the non-left-behind children.

**Figure 3:**
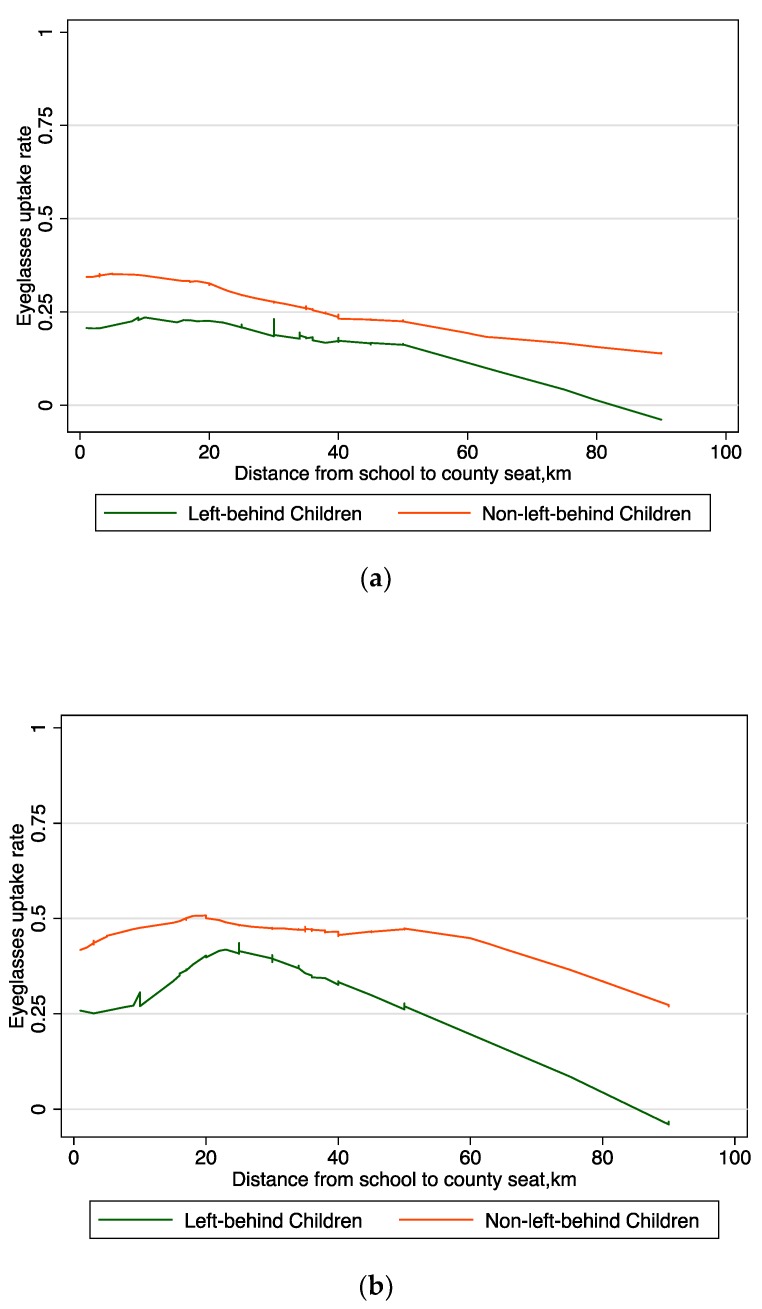
Lowess Plot of eyeglasses uptake rates and distance to county seat among prescription group. (**a**) one month following distribution of prescriptions; (**b**) seven months after distribution of prescriptions.

However, the Lowess plots among the Prescription group in both short term and long term show an opposite pattern—the plot for the left-behind children’s eyeglasses uptake rate was much lower than the non-left-behind children (Figure 3a,b). Furthermore, the uptake rate for the left-behind children in the Prescription group drops sharply as the distance grows. This means the caregivers of left-behind children are much less likely to purchase the eyeglasses, especially when they live far away from the county seat.

**Figure 4:**
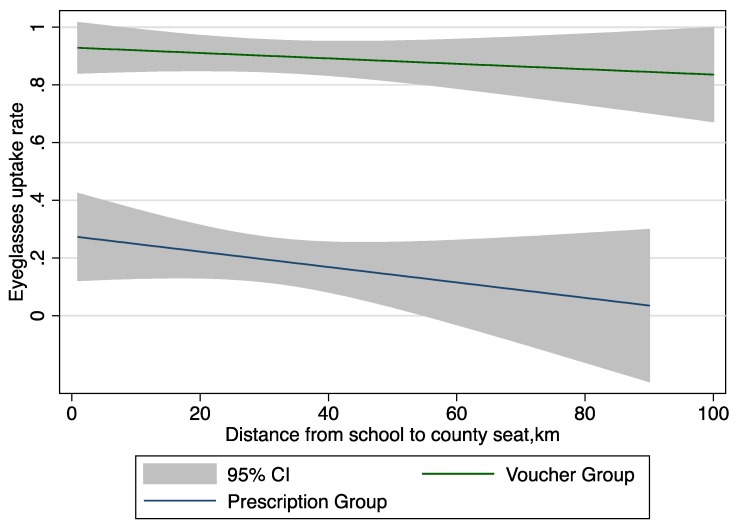
Eyeglasses uptake rates among left-behind children in Voucher and Prescription groups. CI: confidence interval.

But when a subsidy is involved the trends reverses, revealing a higher willingness on the part of left-behind families to travel farther distances to redeem their voucher (Figure 4).

## 4. Discussion

The low rate of eyeglasses usage at baseline accords with similar rates found in other research in rural China [27,31,33]. These low rates, together with the common misapprehensions about the negative effect of eyeglasses on vision, suggest that misinformation may be serving to limit usage of glasses in this context.

The finding that the eyeglasses uptake rate in the Voucher group is significantly higher than in the Prescription group in both short term and long-term echo those reported in other contexts that find subsidies and incentives are important drivers of the uptake of care. Our findings suggest the subsidy may offset the cost of uptake while also potentially signaling a higher value for the service in question. The resulting shift in the cost benefit analysis of individuals leads them to newly favor uptake of services 55 [45,46,47,48].

The eyeglasses usage in Voucher group is significantly higher in both short term and long term, which may be due to the voucher’s capacity to raise individuals’ perceived utility of using eyeglasses. The impact of the voucher highlights the important role subsidies may play not only in the uptake of health services but also compliance with treatment. This finding contrasts with typical “sunk cost effect” considerations regarding subsidized distribution. That is, that subsidies may reduce the psychological effects associated with paying for a product so that goods received for free are less valued and hence less used [47]. Our finding is similar to recent experiments conducted in other developing countries that show no evidence for the psychological sunk cost effect [46,47,49].

Differential impacts on left-behind children provide additional insights into caregiver decision-making. The findings in the Prescription group may indicate that older caregivers (grandparents) do not seek care on the basis of information alone, even if that information is accurate and indicates a problem may exist. Our research thus complements the findings of others suggesting that awareness of a possible health risk alone is insufficient to convince vulnerable groups such as left-behind children and their families to seek out care [45,50]. For example, a summary of evidence from a range of randomized controlled trials on health care services in developing countries, find that the take up of health care services is highly sensitive to price. Liquidity constraints, lack of information, nonmonetary costs, or limited attention also contribute to the limited uptake [45].

The fact that the voucher intervention boosted uptake even more for left-behind children than non-left-behind children is also compelling. This finding suggests that the demand curve for elderly caretakers may be different than that of working age parents. Time, for instance, may be less valuable for elderly caregivers (having retired, they have more of it), so that it costs less for them to uptake care.

One reason for this is the elderly with lower income may be more sensitive to price than distance when seeking health care [51,52,53]. When the health care service is fully subsidized with a voucher, the distance tends to matter less for the left-behind families, i.e., they are willing to travel further in order to obtain a pair of free eyeglasses. As shown in literature, the demand for health care may be affected by the opportunity time cost [54]. Caregivers of left-behind families may have more free time than parents still working and may be more willing to go to the county seat. In this way, left-behind families may be responding more to price than time, in contrast to their non-left-behind counterparts.

Further research is needed to better understand the reason behind the low uptake rate in the Prescription group. While subsidies appear to be effective at raising uptake, a smaller subsidy could have rendered the same outcome in one or both of the groups. A fruitful line of inquiry would therefore be to more fully explore the demand curve for health services among both left-behind and non-left-behind families in China and beyond. This could help determine what factors crowd out the inclination among different types of caregivers to seek certain health solutions for their children in the first place, and in turn open the door to more cost effective polices targeting vulnerable subgroups.

## 5. Conclusions

This study provides new evidence to aid in the design of healthcare policies so that they reach all vulnerable groups in developing countries. The results from a randomized controlled program to provide vision care to rural students in China to better understand how and why left-behind families behave differently to health care interventions.

The findings both confirm the work of others on the importance of subsidies in expanding the reach of care, while also bringing to light the role that differential impacts can have in the design of cost effective health care policy. In the absence of a subsidy, uptake of services in our sample was very low, even if individuals were provided with evidence of a potential problem (an eyeglasses prescription). However, uptake rose dramatically when this information was paired with a subsidy voucher for care (a free pair of eyeglasses). The result complements existing findings that suggest awareness of a possible health risk alone is insufficient to convince vulnerable groups to seek out care [45,50]. What is more, the findings confirm the broad value of subsidies when seeking to raise uptake of underutilized health services.

The analysis of the differential impacts of the subsidy on left-behind children contributes another layer of understanding to this consensus. Left-behind children who receive an eyeglasses voucher were not only more likely to redeem it, but also more likely to use the eyeglasses both in the short term and long term. In other words, in terms of uptake of care and compliance with treatment, the voucher program benefitted left-behind students more than non-left-behind students. This finding is similar to recent programs conducted in other developing countries that show no evidence for the psychological sunk cost effect of subsidized distribution [46,47,49]. This trend may occur because left-behind families are more sensitive to price than distance when seeking health care.

More broadly, the findings on the differential impacts of the voucher programs highlight the existence of systematically different patterns of response among large subpopulations like left-behind children, suggesting that groups may require tailored solutions to reach health policy goals. Further understanding the potential for tailored solutions of this kind may have wide ranging implications for health policy in primary care, infectious disease control, and chronic disease management.

## Figures and Tables

**Table 1:** Balance check of sample students baseline characteristics across experimental groups.

Variables	Prescription Group	Voucher Group	Difference	*p*-Value
	n = 1036	n = 988		
	(1)	(2)	(2)−(1)	
1. Age (Years)	10.546	10.513	−0.033	0.673
	(1.109)	(1.109)		
2. Male, 1 = yes	0.499	0.480	−0.019	0.379
	(0.500)	(0.500)		
3. Boarding at school, 1 = yes	0.227	0.185	−0.042	0.377
	(0.419)	(0.389)		
4. Grade four, 1 = yes	0.404	0.385	−0.020	0.380
	(0.491)	(0.487)		
5. Owns eyeglasses, 1 = yes	0.139	0.140	0.001	0.973
	(0.346)	(0.347)		
6. Believe eyeglasses harm vision, 1 = yes	0.415	0.364	−0.051	0.114
	(0.493)	(0.481)		
7. Visual acuity of worse eye (LogMAR)	0.647	0.621	−0.027	0.104
	(0.215)	(0.210)		
8. Father has high school education or above, 1 = yes	0.157	0.134	−0.024	0.229
	(0.364)	(0.340)		
9. Mother has high school education or above, 1 = yes	0.099	0.078	−0.021	0.172
	(0.299)	(0.268)		
10. At least one family member wears glasses, 1 = yes	0.347	0.325	−0.022	0.322
	(0.476)	(0.469)		
11. Household assets (index)	−0.053	−0.064	−0.011	0.923
	(1.275)	(1.280)		
12. Distance from school to the county seat (Km)	34.871	32.212	−2.659	0.515
	(19.758)	(23.826)		
13. Both parents at home, 1 = yes	0.509	0.487	−0.022	0.511
	(0.500)	(0.500)		
14. Both parents migrated, 1 = yes	0.100	0.124	0.024	0.138
	(0.301)	(0.330)		

Data source: baseline survey. The Prescription group only received eyeglass prescriptions. In the Voucher group, vouchers for free eyeglasses were redeemable in the county seat.

**Table 2:** Test of differential attrition between short term and long- term surveys.

Variables	(1)	(2)	(3)	(4)	(5)	(6)	(7)	(8)	(9)	(10)	(11)	(12)	(13)	(14)
	Age (Years)	Male, 1 = yes	Boarding at School, 1 = yes	Grade Four, 1 = yes	Owns Eyeglasses, 1 = yes	Believe Eyeglasses Harm Vision, 1 = yes	Visual Acuity of Worse Eye (LogMAR)	Father Has High School Education or Above, 1 = yes	Mother Has High School Education or Above, 1 = yes	At Least One Family Member Wears Glasses, 1 = yes	Household Assets (Index)	Distance from School to the County Seat. km	Both Parents at Home, 1 = yes	Both Parents Migrated, 1 = yes
Panel A: Balance between Voucher and Prescription group accounting for attrition in short term														
Voucher Group Dummy	−0.038	−0.019	−0.039	−0.020	0.001	−0.044	−0.027	−0.020	−0.019	−0.022	−0.014	−2.546	−0.022	0.021
	(0.080)	(0.022)	(0.047)	(0.023)	(0.020)	(0.032)	(0.017)	(0.019)	(0.015)	(0.022)	(0.111)	(4.089)	(0.034)	(0.016)
Attrition short term	0.162	0.227 **	0.108	−0.016	−0.028	0.313 **	−0.014	0.179	0.125	0.156	0.116	4.146	−0.122	−0.102 ***
	(0.262)	(0.092)	(0.131)	(0.131)	(0.063)	(0.120)	(0.064)	(0.167)	(0.082)	(0.117)	(0.730)	(5.371)	(0.097)	(0.011)
Voucher × Short term attrition	0.273	0.003	−0.177	−0.016	0.006	−0.384 **	0.035	−0.195	−0.144	−0.007	0.174	−6.516	−0.014	0.155
	(0.341)	(0.165)	(0.153)	(0.183)	(0.104)	(0.166)	(0.071)	(0.182)	(0.101)	(0.163)	(0.804)	(7.865)	(0.150)	(0.100)
Constant	10.544 ***	0.495 ***	0.225 ***	0.405 ***	0.139 ***	0.410 ***	0.648 ***	0.154 ***	0.097 ***	0.344 ***	−0.055	34.799 ***	0.511 ***	0.102 ***
	(0.059)	(0.016)	(0.035)	(0.016)	(0.014)	(0.021)	(0.011)	(0.013)	(0.011)	(0.015)	(0.081)	(2.617)	(0.026)	(0.011)
*R^2^*	0.002	0.004	0.004	0.000	0.000	0.006	0.004	0.003	0.003	0.002	0.001	0.004	0.002	0.003
Panel B: Balance between Voucher and Prescription group accounting for attrition in long term														
Voucher Group Dummy	−0.034	−0.024	−0.042	−0.020	0.004	−0.046	−0.027	−0.023	−0.023	−0.026	−0.013	−2.624	−0.018	0.022
	(0.078)	(0.022)	(0.046)	(0.023)	(0.020)	(0.032)	(0.017)	(0.020)	(0.016)	(0.023)	(0.114)	(4.034)	(0.035)	(0.016)
Attrition long term	−0.048	−0.140	0.203 ***	0.083	0.076	0.041	−0.005	0.088	0.085	−0.014	−0.134	5.736	−0.525 ***	0.272 ***
	(0.258)	(0.092)	(0.071)	(0.087)	(0.063)	(0.098)	(0.043)	(0.072)	(0.068)	(0.084)	(0.294)	(4.114)	(0.026)	(0.085)
Voucher × Long term attrition	0.021	0.148	−0.041	−0.026	−0.094	−0.116	0.017	−0.049	0.012	0.083	0.082	−2.191	0.018	−0.020
	(0.345)	(0.117)	(0.104)	(0.118)	(0.077)	(0.123)	(0.051)	(0.103)	(0.091)	(0.106)	(0.364)	(6.322)	(0.035)	(0.124)
Constant	10.548 ***	0.503 ***	0.221 ***	0.402 ***	0.137 ***	0.414 ***	0.648 ***	0.155 ***	0.097 ***	0.348 ***	−0.048	34.688 ***	0.525 ***	0.092 ***
	(0.058)	(0.016)	(0.035)	(0.017)	(0.014)	(0.021)	(0.012)	(0.014)	(0.012)	(0.016)	(0.083)	(2.607)	(0.026)	(0.012)
*R^2^*	0.000	0.002	0.010	0.001	0.001	0.003	0.004	0.002	0.005	0.001	0.000	0.005	0.038	0.026

All estimates adjusted for clustering at the school level. Robust standard errors reported in parentheses; *n* = 2024; *** *p* < 0.01, ** *p* < 0.05, * *p* < 0.1.

**Table 3:** Differences in left-behind children and non-left-behind children with poor vision at the time of baseline.

Variables	Non-Left-Behind	Left-Behind	Difference	*p*-Value
	n = 1797	n = 227		
	(1)	(2)	(2)−(1)	
1. Age (Years)	10.530	10.535	0.006	0.940
	(1.087)	(1.276)		
2. Male, 1 = yes	0.494	0.458	−0.035	0.314
	(0.500)	(0.499)		
3. Boarding at school, 1 = yes	0.206	0.212	0.006	0.827
	(0.405)	(0.410)		
4. Grade four, 1 = yes	0.390	0.436	0.047	0.176
	(0.488)	(0.497)		
5. Owns eyeglasses, 1 = yes	0.141	0.128	−0.013	0.593
	(0.348)	(0.335)		
6. Believe eyeglasses harm vision, 1 = yes	0.393	0.370	−0.023	0.507
	(0.489)	(0.484)		
7. Visual acuity of worse eye (LogMAR)	0.634	0.637	0.003	0.850
	(0.211)	(0.224)		
8. Father has high school education or above, 1 = yes	0.145	0.150	0.005	0.855
	(0.352)	(0.358)		
9. Mother has high school education or above	0.081	0.150	0.069 ***	0.001
	(0.273)	(0.358)		
10. At least one family member wears glasses	0.337	0.332	−0.005	0.881
	(0.473)	(0.472)		
11. Household assets (Index)	−0.019	−0.364	−0.345 ***	0.000
	(1.273)	(1.268)		
12. Distance from school to the county seat. km	33.866	31.247	−2.620	0.089
	(21.868)	(21.823)		

Data source: baseline survey. All tests account for clustering at the school level. *** *p* < 0.01, ** *p* < 0.05, * *p* < 0.1.

**Table 4:** Average impact of providing voucher on eyeglasses uptake and usage (irrespective of left behind status).

Variables	Eyeglasses Uptake				Eyeglasses Usage			
	Short Term		Long Term		Short Term		Long Term	
	(1)	(2)	(3)	(4)	(5)	(6)	(7)	(8)
	Unadjusted	Adjusted	Unadjusted	Adjusted	Unadjusted	Adjusted	Unadjusted	Adjusted
Voucher	0.606 ***	0.611 ***	0.447 ***	0.454 ***	0.446 ***	0.454 ***	0.246 ***	0.265 ***
	(0.022)	(0.020)	(0.024)	(0.024)	(0.025)	(0.022)	(0.027)	(0.025)
Controls		Yes		Yes		Yes		Yes
Constant	0.262 ***	0.091	0.456 ***	0.074	0.227 ***	−0.080	0.379 ***	0.043
	(0.015)	(0.113)	(0.019)	(0.126)	(0.016)	(0.122)	(0.019)	(0.153)
Observations	1989	1980	1950	1941	1989	1980	1950	1941
*R* ^2^	0.411	0.527	0.262	0.332	0.258	0.390	0.122	0.231
Mean in prescription group	0.248		0.427		0.210		0.345	

Columns (1) to (8) show coefficients on treatment group indicators estimated by ordinary least squares (OLS). Columns (1) to (4) report estimates impact of providing voucher on eyeglasses uptake. Columns (4) to (8) report estimates impact of providing voucher on eyeglasses usage. Columns (1) (2) (5) and (6) report the short-term follow up in one month after initial voucher distribution. Columns (3) (4) (7) and (8) report estimates for the long-term follow up in seven months after initial voucher or prescription distribution. Sample sizes are less than the full sample due to observations missing at least one regressor. Standard errors clustered at school level are reported in parentheses. All regressions control for randomization strata indicators. *** Significant at the 1% level.

**Table 5:** Heterogeneous impact of providing a subsidy voucher on left-behind children eyeglasses uptake and usage.

	Eyeglasses Uptake				Eyeglasses Usage			
	Short term		Long term		Short term		Long term	
	(1)	(2)	(3)	(4)	(5)	(6)	(7)	(8)
	Unadjusted	Adjusted	Unadjusted	Adjusted	Unadjusted	Adjusted	Unadjusted	Adjusted
1. Voucher Group	0.597 ***	0.603 ***	0.435 ***	0.442 ***	0.437 ***	0.445 ***	0.233 ***	0.251 ***
	(0.022)	(0.020)	(0.025)	(0.025)	(0.026)	(0.024)	(0.029)	(0.026)
2. Left-behind children	−0.065 *	−0.050 *	−0.109 **	−0.098 **	−0.056	−0.044	−0.131 ***	−0.120 ***
	(0.034)	(0.028)	(0.051)	(0.049)	(0.036)	(0.033)	(0.045)	(0.044)
3. Voucher * Left-behind	0.084 **	0.068 *	0.131 **	0.118 **	0.076	0.072	0.146 **	0.137 **
	(0.042)	(0.040)	(0.056)	(0.056)	(0.054)	(0.054)	(0.063)	(0.062)
Baseline controls		YES		YES		YES		YES
Constant	0.269 ***	0.096	0.466 ***	0.080	0.233 ***	−0.075	0.391 ***	0.050
	(0.015)	(0.113)	(0.020)	(0.125)	(0.017)	(0.122)	(0.020)	(0.151)
Observations	1989	1980	1950	1941	1989	1980	1950	1941
*R* ^2^	0.411	0.527	0.264	0.333	0.258	0.390	0.124	0.232

Columns (1) to (8) show coefficients on treatment group indicators estimated by OLS. Columns (1) to (4) report estimates impact of providing voucher on eyeglasses uptake. Columns (4) to (8) report estimates impact of providing voucher on eyeglasses usage. Columns (1) (2) (5) and (6) report the short-term follow up in one month after initial voucher distribution. Columns (3) (4) (7) and (8) report estimates for the long-term follow up in seven months after initial voucher or prescription distribution. Sample sizes are less than the full sample due to observations missing at least one regressor. Standard errors clustered at school level are reported in parentheses. All regressions control for randomization strata indicators. *** Significant at the 1% level. ** Significant at the 5%. * Significant at the 10% level.
